# Effect of Moving Tactile Stimuli to Sole on Body Sway During Quiet Stance

**DOI:** 10.3390/brainsci15121282

**Published:** 2025-11-28

**Authors:** Taku Kawasaki, Yasushi Sawaguchi, Koichi Hiraoka

**Affiliations:** 1 Graduate School of Rehabilitation Science, Osaka Metropolitan University, 132-1 Morinomiya 2-chome, Osaka 536-8525, Japan; su24361a@st.omu.ac.jp; 2 Graduate School of Comprehensive Rehabilitation, Osaka Prefecture University, Habikino 583-8555, Japan; yasushi.19900720@gmail.com

**Keywords:** moving tactile stimuli, quiet stance, body sway, center of pressure, intermodal reweighting, vision, tactile sensation

## Abstract

**Objectives:** This study examined whether moving tactile stimuli applied to the soles along the anterior–posterior (AP) and medial–lateral (ML) axes induce body sway during quiet stance. **Methods:** Fifteen healthy participants in quiet stance received plantar moving tactile stimuli along the AP or ML axis under occluded and unoccluded vision conditions. **Results:** The center of pressure (COP) along the ML axis was dependent on the phase of moving tactile stimuli along the ML axis under occluded vision condition. The direction of body sway was opposite to the stimulus loci. The total COP displacement during moving tactile stimuli along the AP axis was larger than that without stimuli, particularly under occluded vision conditions. **Discussion:** Humans likely perceive body sway towards the stimulated side. Based on this, when humans perceive body sway along the ML axis, they compensate for it by swaying the body in the direction contralateral to the stimulated side. Body sway along the ML axis, in accordance with the plantar loci receiving input of moving tactile sensation along the same axis, becomes apparent under occluded vision condition. Through intermodal reweighting, the contribution of tactile sensation to the control of body sway along the AP axis increases to compensate for the lack of visual input regulating body sway along this axis.

## 1. Introduction

In humans, quiet stance is maintained through various sensory modalities, such as vestibular, proprioceptive, visual, and plantar tactile sensations [1], and the only contact points between the body and the ground are the soles of the feet [2]. The soles have cutaneous sensory receptors that enable the determination of weight loading on the ground based on changes in the tactile input at each receptor locus. Thus, plantar cutaneous sensation plays a crucial role in perceiving body weight distribution and body sway during quiet stance. Previous studies have shown that sensitivity to plantar vibration and tactile stimuli was significantly reduced in older adults compared with young adults [3] and that aging deteriorated postural control relying on plantar tactile perception [4]. Accordingly, age-related changes in postural control during quiet stance are largely related to the tactile sensation of the soles.

Previous findings regarding the contribution of the stationary tactile sensation of the soles to body sway during quiet stance also support this view. For example, cutaneous anesthesia applied to the soles increased the displacement and velocity of the center of pressure (COP) during quiet stance under occluded vision condition [5]. COP displacement during quiet stance increased when the tactile sensation of the soles was masked with an ischemic nerve block [6]. The acceleration of lumbar deviation during quiet stance was greater in patients with diabetic peripheral neuropathy who suffered from impaired tactile sensation [7]. The recovery from leftward body sway during quiet stance was greater when the tactile sensation of the soles was masked [8]. Applying tactile stimuli to the soles while masking tactile sensation increased COP displacement and velocity during quiet stance [9].

Humans maintain balance during quiet stance through the perception of weight distribution on the soles over time enabled by tactile sensation. Thus, the change in the loci of tactile sensation likely mediates the perception of the change in weight distribution during quiet stance. Artificial moving tactile stimuli applied to specific loci of the soles induce the perception of moving tactile sensation. In response to a moving stimulus, three types of cutaneous mechanoreceptors convey distinct signals [10]: Merkel endings are densely distributed and possess high spatial resolution, encoding the spatiotemporal patterns of the shape and position of stimuli moving across the skin; Meissner corpuscles are highly sensitive to temporal changes and perceive continuously moving stimuli through sequential activation; Ruffini endings detect skin stretch and transmit deformation patterns that depend on the direction of movement. These signals are represented through isomorphic coding, in which adjacent receptors fire sequentially in response to tactile stimuli moving across the skin, thereby encoding the direction of motion in a spatiotemporal manner. Through this coding mechanism, the application of moving tactile stimuli to the soles induces the illusory perception of temporal changes in weight distribution. Based on this, in the present study, the change in body sway induced by moving tactile stimuli applied to the soles was examined.

Previous studies investigated the cortical activity during the moving tactile stimuli to the body parts [10,11]. Moving tactile stimuli to the body parts induced cortical activity in the primary sensory cortex, secondary sensory cortex, posterior parietal cortex, and hMT+/V5 [12,13,14,15,16]. Based on these previous findings, the application of moving tactile stimuli to the soles may influence postural control during quiet stance via the cortical response to stimuli.

Supporting this view, the COP rhythmically displaced along the medial-lateral (ML) axis along with the phase of the change in the loci of the stimuli during moving tactile stimuli [17]. In this study, the loci of the soles were stimulated so as to mimic the change in weight distribution during gait by applying moving stimuli along both the anterior–posterior (AP) and ML axes over time, but it was not determined whether the observed effect was due to the moving tactile stimuli along the ML or AP axis. In the present study, we aimed to provide further insights into the above findings by separately examining the body sway during quiet stance induced by tactile stimuli moving along the AP axis and that induced by stimuli moving along the ML axis, which allowed us to distinguish the effects of tactile stimuli along the two axes. Tactile receptors are sensitive to the direction of the temporal change in tactile stimulus location [10]. Based on this, we hypothesized that the effects of tactile stimuli moving along the ML and AP axes are different.

## 2. Materials and Methods

### 2.1. Participants

The participants were 15 healthy males aged 31.7 ± 7.8 years. Only males were recruited to exclude the variability in postural responses caused by gender differences in physical characteristics, motor performance [18,19], and postural control [20] and to minimize the inter-individual variability in postural control, which has been shown to be greater in females than in males [21]. The required sample size was estimated using G*Power 3.1.9.6. To achieve a statistical power of 0.80 with an alpha level of 0.05, a total of 12 participants were required to detect a large effect (f = 0.4) in two-way repeated-measures ANOVA. The participants had no history of neurological or musculoskeletal diseases. Two of our participants were left-footed, twelve were right-footed, and one was ambidextrous, according to the revised version of the Waterloo Footedness Questionnaire [22,23]. We obtained written consent from all participants before they participated in the study. The experiment was approved by the ethics committee of Osaka Prefecture University (approval number: 2021-110).

### 2.2. Apparatus

**Figure 1:**
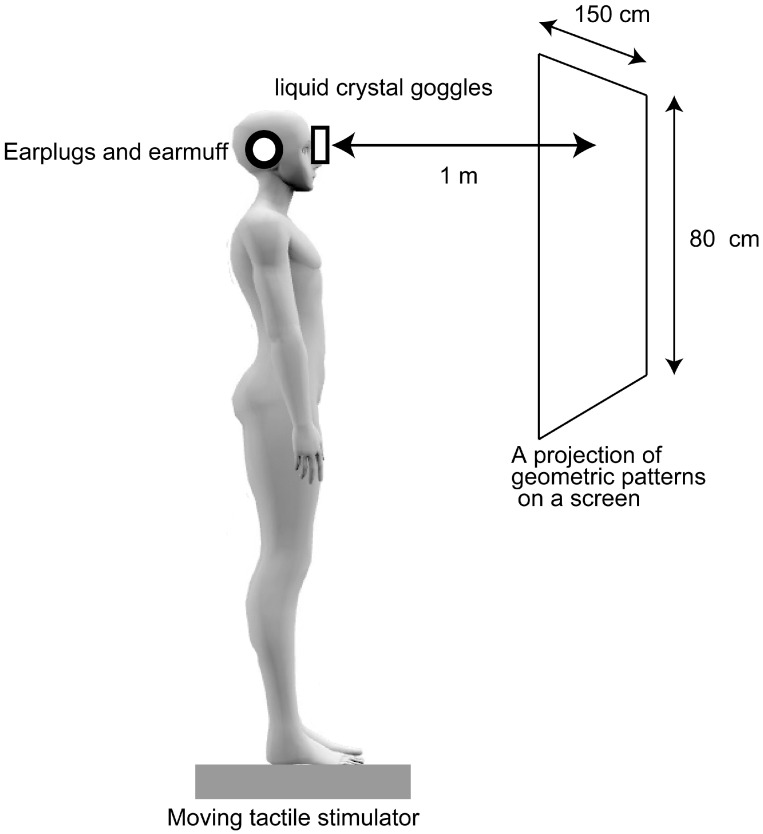
Experimental setup.

**Figure 2:**
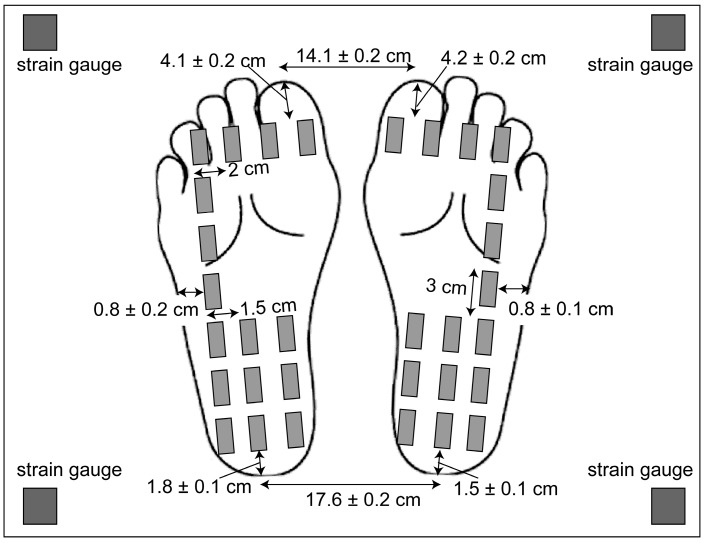
The loci of the vibration devices and feet position on the moving tactile stimulator. The distances between the vibration devices are shown. The other numbers indicate means and the standard errors of the means.

The setup of the experiment is shown in Figure 1. The participants wore liquid crystal goggles to occlude their vision (T.K.K.2275; Takei Kiki, Tokyo, Japan), as well as earplugs and an earmuff over the ears to minimize unnecessary auditory input. A screen 80 cm in height and 150 cm in width showing geometric patterns was placed on a vertical wall 1 m in front of the participants. A moving tactile stimulator (S-20008; Takei Kiki, Tokyo, Japan) was placed on the ground [17,24]. The device consisted of 16 vibrators attached to the support surface of each foot (Figure 2), excluding the area under the medial longitudinal arch, which did not come into contact with the ground. Each vibrator (linear actuator) vibrated at the frequency of 147 Hz. Vibratory stimuli between 100 and 200 Hz predominantly excite the Pacinian corpuscles, which are tuned to high-frequency skin vibrations [25,26,27], while peak muscle spindle response is induced by a vibration frequency of about 80–100 Hz [28,29]. Thus, cutaneous receptors can be selectively activated by using these vibration frequencies. The linear actuator created motion in a straight line, had a size of 11.2 mm × 14.0 mm × 2.6 mm, and was driven by a 5 V rated power supply. The vibration devices were placed under the soles while the participant maintained a quiet stance. A strain gauge was placed at each corner of the rectangular platform to measure strain and subsequently calculate the COP.

The COP is expressed as

$$COPx(mm)=\frac{-V1-V2+V3+V4}{V1+V2+V3+V4}\;\frac{275}{2}$$

$$COPy(mm)=\frac{V1-V2-V3+V4}{V1+V2+V3+V4}\;\frac{275}{2}$$

where *V* denotes the voltage output from each strain gauge.

### 2.3. Procedure

The participants maintained a quiet stance on the support surface of the moving tactile stimulator, with their arms resting comfortably at the sides. We did not instruct participants to sway their bodies in accordance with the moving tactile stimuli, because we aimed to evaluate involuntary body sway upon moving tactile stimuli. The position of the feet was finely adjusted immediately before each session so that all the vibration devices were under the soles. The foot position relative to the positions of the vibration devices is shown in Figure 1 [17]. The participants gazed at a geometric pattern presented on a wall 1 m in front of them [17]. In the sessions with visual occlusion (NV), the glass of the goggles was opaque, so that vision was occluded, while in the sessions without visual occlusion (V), the glass was transparent, so that vision was unoccluded. Tactile stimuli moving along the AP or ML axis were administered during the stimulus sessions (AP and ML sessions) but not during the control session. In total, we conducted six sessions: NV and V for the control and stimulus sessions (AP and ML sessions). The COP displacements in the control and stimulus sessions were compared to examine the effect of the tactile stimuli, while the comparison of NV and V conditions was conducted to determine whether the effect of moving tactile stimuli is masked under unoccluded vision condition, given that intermodal reweighting occurs when one modality is masked [8]. The order of these six sessions was random for each participant, and the interval between sessions was four minutes.

### 2.4. Moving Tactile Stimuli

**Figure 3:**
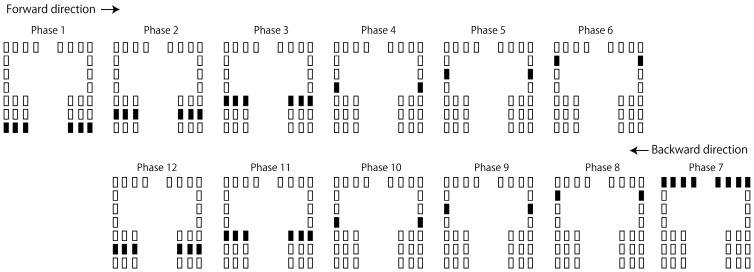
The protocol of tactile stimuli along the AP axis. Each schematic illustrates the activity of the vibration devices in each phase (83 ms per phase). The rectangles denote the vibration devices, with filled rectangles indicating the active devices in each phase.

Tactile stimuli moving along the AP axis were administered in the AP session (Figure 3). There were twelve stimulation phases per cycle, with each phase lasting 83 ms. In each cycle, the stimuli started from the heels and moved toward the toes (from phases 1 to 7), and when it reached phase 7, it moved back toward the heels (from phases 7 to 12). Therefore, the frontal loci were stimulated in phase 7 and the posterior loci in phase 1. In this session, there were 40 stimulation cycles, each lasting 994 ms. According to interviews with four participants, they all subjectively perceived that the tactile stimuli moved along the AP axis.

**Figure 4:**
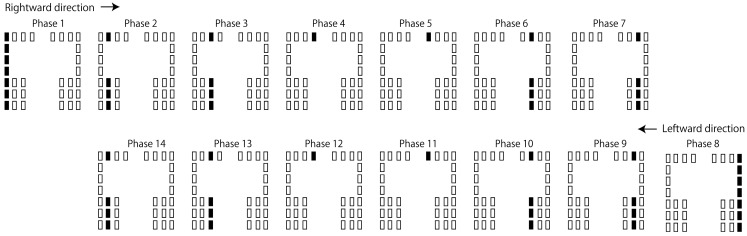
The protocol of tactile stimuli along the ML axis. Each schematic illustrates the activity of the vibration devices in each phase (71 ms per phase). The rectangles denote the vibration devices, with filled rectangles indicating the active devices in each phase.

Tactile stimuli moving along the ML axis were administered in the ML session (Figure 4). There were fourteen stimulation phases per cycle, with each phase lasting 71 ms. In each cycle, the stimulus started from the leftmost column and moved to the right (from phases 1 to 8), and when it reached the rightmost column (phase 8), it moved back to the left (from phases 8 to 14). Therefore, the leftmost loci were stimulated in phase 1 and the rightmost loci in phase 8. Each cycle lasted 996 ms, with 40 cycles per session. According to the interviews with four participants, they all subjectively perceived that the tactile stimuli moved along the ML axis.

### 2.5. Data Analysis

The COP displacement along the AP axis is denoted by COPy and that along the ML axis by COPx. The total COP displacement in each session was calculated to represent the amount of body sway by estimating the displacement every 1 ms and summing all measurements within the time window. The mean COP in each session was calculated to represent the body position. The z-scores of the mean COP and total COP displacement were calculated [17]. The mean COP or total COP displacement in each session was subtracted from the overall average across sessions and then divided by the standard deviation of the session-wise values across sessions. A positive z-score of the COP indicates a displacement toward the right or in the anterior direction, while a negative one indicates a displacement toward the left or in the posterior direction. The mean COP in each phase of the stimulation cycle and its z-score were also calculated. Body sway with a dominant frequency below 1 Hz is generally attributed to control through the ankle strategy, while that above 1 Hz reflects control through the hip strategy [30]. Based on this, the median frequency of COP displacement was measured for each stimulation condition to determine whether the body sway during stimuli was predominantly controlled via the ankle or hip strategy.

Two-way repeated measures ANOVAs were conducted to test the main effect of the stimuli (control, AP, and ML) and vision (V and NV) on the z-scores of the mean COP and total COP displacement. In addition, repeated measures ANOVAs were conducted to test the main effect of the stimulation phases on the COP. The result of the Greenhouse–Geisser correction was reported whenever Mauchly’s test of sphericity was significant. When there was a significant interaction between the main effects, we conducted a test of the simple main effects. The Bonferroni test was conducted for multiple comparisons, and the alpha level was 0.05. All statistical analyses were carried out using Excel Tokei ver. 4.09 (Social Survey Research Information, Tokyo, Japan). All data in the Results section are expressed as means and standard errors of the mean.

## 3. Results

### 3.1. COP in Each Phase of Moving Tactile Stimuli

**Figure 5:**
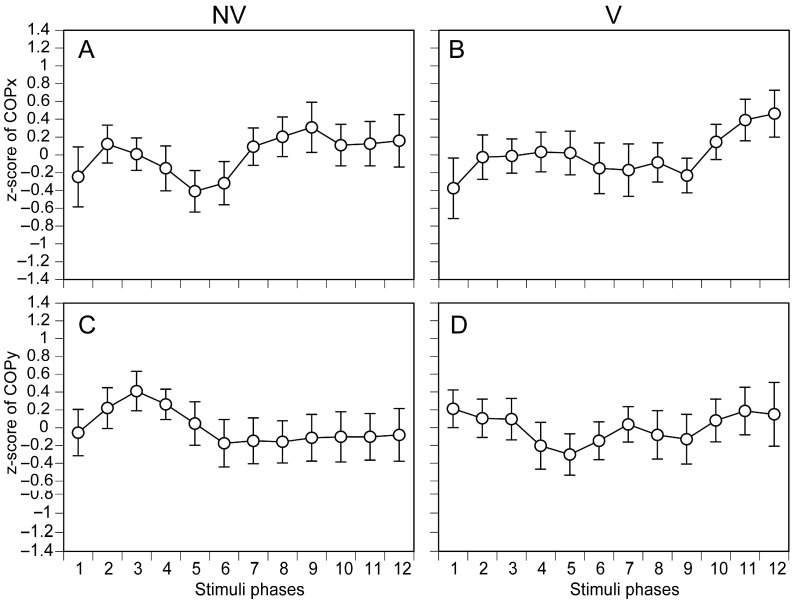
Phase-dependent COP displacement during moving tactile stimuli along the AP axis. COPx under NV (**A**) and under V (**B**), and COPy under NV (**C**) and under V (**D**) are presented. The data points represent means, and the error bars represent the standard errors of the means.

The COPx and COPy measured in each phase of moving tactile stimuli along the AP axis are shown in Figure 5. There were no significant main effects of the stimulation phase on COPx under NV [F (4.886, 68.407) = 0.758, *p* = 0.580] (Figure 5A) and V [F (2.877, 40.282) = 0.871, *p* = 0.460] (Figure 5B) conditions nor on COPy under NV [F (3.093, 43.301) = 0.541, *p* = 0.662] (Figure 5C) and V [F (3.109, 43.529) = 0.403, *p* = 0.758] (Figure 5D) conditions.

**Figure 6:**
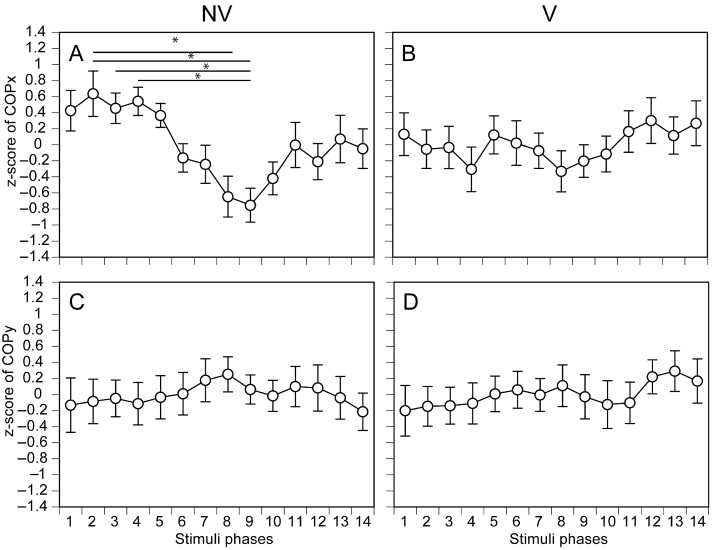
Phase-dependent COP displacement during moving tactile stimuli along the ML axis. The COPx under NV (**A**) and V (**B**), and COPy under NV (**C**) and V (**D**) are presented. The data points represent means, and the error bars represent the standard errors of the means. The asterisks indicate significant differences between phases according to the multiple comparison test (Bonferroni test, *p* < 0.05).

The COPx measured in each phase of moving tactile stimuli along the ML axis is shown in Figure 6A,B. There was no significant main effect of the stimulation phase under V condition [F (3.499, 48.986) = 0.563, *p* = 0.668] (Figure 6B). There was, however, a significant main effect of the stimulation phase under NV condition [F (4.477, 62.673) = 3.359, *p* = 0.012] (Figure 6A). A multiple comparison test revealed that the COPx measured in phase 2 was significantly deviated rightward compared with that in phase 8 (*p* = 0.020) or phase 9 (*p* = 0.006). Similarly, the value measured in phase 3 was significantly deviated rightward compared with that in phase 9 (*p* = 0.044), as did that in phase 4 (*p* = 0.017). The COPy measured in each phase of moving tactile stimuli along the ML axis is shown in Figure 6C,D. In this case, there were no significant main effects of the stimulation phase under NV [F (3.583, 50.156) = 0.224, *p* = 0.908] (Figure 6C) and V [F (3.235, 45.287) = 0.329, *p* = 0.819] (Figure 6D) conditions.

### 3.2. Frequency of COP Displacement

**Figure 7:**
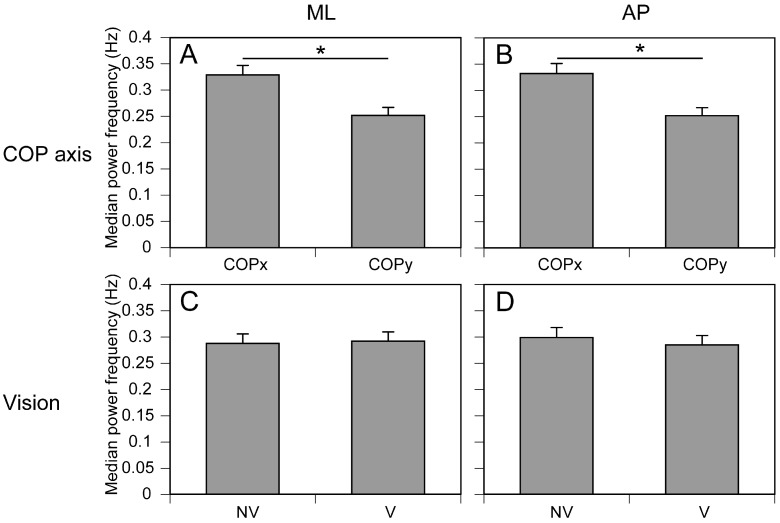
Main effects on median frequency of COP displacement. The median frequencies during the moving tactile stimuli in the ML axis are shown in (**A**,**C**), and those in the AP axis are shown in (**B**,**D**). The upper panels (**A**,**B**) present the effect of COP axis and the lower panels (**C**,**D**) present the effect of vision. The bars represent means, and the error bars represent the standard errors of the means. Each asterisk indicates a significant main effect of the body sway axis (*p* < 0.05).

The frequency of COP displacement is shown in Figure 7. The mean medial frequency of COPx was 0.332 ± 0.019 Hz, and that of COPy was 0.252 ± 0.015 Hz during moving tactile stimuli along the AP axis. The median frequency of COPx was significantly greater than that of COPy [F (1, 14) = 27.272, *p* < 0.001] (Figure 7B). There were no significant main effect of vision [F (1, 14) = 0.544, *p* = 0.473] (Figure 7D) nor significant interaction between the main effects [F (1, 14) = 0.0.17, *p* = 0.899]. The mean medial frequency of COPx was 0.329 ± 0.018 Hz and that of COPy was 0.252 ± 0.015 Hz during moving tactile stimuli along the ML axis. The median frequency of COPx was significantly greater than that of COPy [F (1, 14) = 16.358, *p* = 0.001] (Figure 7A). There were no significant main effects on vision [F (1, 14) = 0.072, *p* = 0.793] (Figure 7C) nor significant interactions between the main effects [F (1, 14) = 0.002, *p* = 0.962].

### 3.3. Total COP Displacement

**Figure 8:**
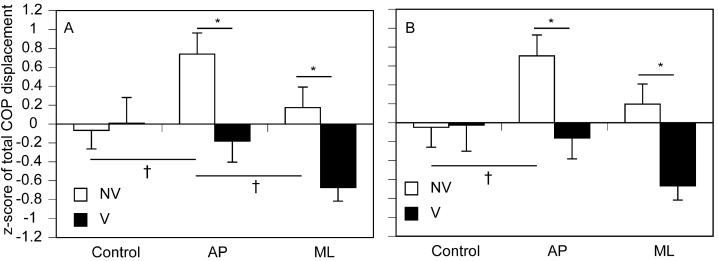
Total COP displacement. The COPx is presented in (**A**) and COPx is presented in (**B**). The bars represent means, and the error bars represent the standard errors of the means. The asterisks indicate significant differences between NV and V conditions according to tests of simple main effects (*p* < 0.05). The daggers indicate significant differences between stimulation conditions according to multiple comparison tests (Bonferroni test; *p* < 0.05).

The total COPx is shown in Figure 8A. There was a significant main effect of vision [F (1, 14) = 5.024, *p* = 0.042] but not of stimuli [F (2, 28) = 2.768, *p* = 0.080], and there was a significant interaction between vision and stimuli [F (2, 28) = 3.603, *p* = 0.041]. The simple main effect of vision was insignificant in the control [F (1, 36) = 0.047, *p* = 0.829]. However, the total COPx under NV condition was significantly greater than that under V condition during moving tactile stimuli along the AP [F (1, 36) = 7.049, *p* = 0.012] and ML [F (1, 36) = 5.956, *p* = 0.020] axes in accordance with test of simple main effect. There was a simple main effect of stimuli under NV condition [F (2, 56) = 3.660, *p* = 0.032]. A multiple comparison test revealed that the total COPx during moving tactile stimuli along the AP axis was significantly greater than both that under control condition and that during stimuli along the ML axis under NV condition (*p* < 0.05). In contrast, such a significant simple main effect was absent under V condition.

The total COPy is shown in Figure 8B. There was a significant main effect of vision [F (1, 14) = 4.783, *p* = 0.046] but not of stimuli [F (2, 28) = 2.450, *p* = 0.105], and there was a significant interaction between vision and stimuli [F (2, 28) = 3.467, *p* = 0.045]. The simple main effect of vision was insignificant in the control [F (1, 33) = 0.004, *p* = 0.948]. In contrast, the total COPy under NV condition was significantly greater than that under V condition during moving tactile stimuli along the AP [F (1, 33) = 6.370, *p* = 0.017] and ML [F (1, 33) = 6.289, *p* = 0.017] axes in accordance with test of simple main effect. There was a simple main effect of stimuli under NV condition [F (2, 54) = 3.254, *p* = 0.046]. A multiple comparison test revealed that the total COPy during moving tactile stimuli along the AP axis was significantly greater than that under control NV condition (*p* < 0.05), but such a significant simple main effect was absent under V condition.

### 3.4. Mean COP

**Figure 9:**
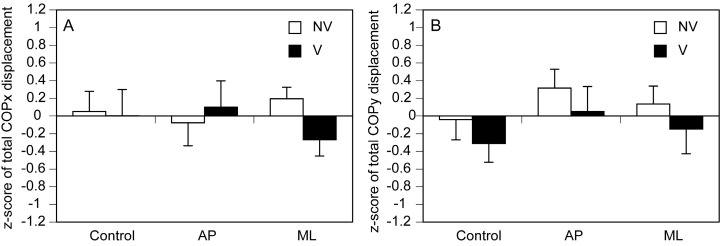
Mean COP displacement. The COPx is presented in (**A**) and COPx is presented in (**B**). The bars represent means, and the error bars represent the standard errors of the means.

The mean COPx is shown in Figure 9A. There were no significant main effects of vision [F (1, 14) = 0.396, *p* = 0.539] nor of stimuli [F (2, 28) = 0.027, *p* = 0.974], and there were no significant interaction between vision and stimuli [F (2, 28) = 0.777, *p* = 0.469]. The mean COPy is shown in Figure 9B. Similarly, no significant main effects of vision [F (1, 14) = 1.001, *p* = 0.334] nor of stimuli [F (2, 28) = 1.047, *p* = 0.365] and no significant interaction between vision and stimuli [F (2, 28) = 0.001, *p* = 0.999] were observed.

## 4. Discussion

### 4.1. Summary of Main Findings

The present study demonstrated that moving tactile stimuli to the soles along the ML axis induced phase-dependent lateral body sway during quiet stance. Specifically, the direction of body sway was opposite to the stimulus. Such escaping behavior has been observed upon the application of static tactile stimuli [29,31]. Nevertheless, the present study is the first to show that the body sways in accordance with the phase of tactile stimuli moving along the ML axis. These findings extend previous knowledge of the effects of moving tactile stimuli on body sway during quiet stance by dissociating the contributions of ML and AP tactile stimuli to postural control mechanisms.

### 4.2. Phase-Dependent COP Changes

On the one hand, the COPx values measured in the various phases of moving tactile stimuli along the ML axis differed. This means that the body sways along the ML axis in accordance with the phase of a tactile stimulus moving along the same axis. On the other hand, there was no such phase-dependent change in the COP when stimuli were applied along the AP axis. A previous study reported phase-dependent body sway under moving tactile stimuli during quiet stance and observed that the COP along the ML axis was rhythmically displaced in accordance with the moving tactile stimulation phase, mimicking the weight distribution changes during human gait [17]. To mimic the weight distribution change over the soles during gait, tactile stimuli need to be applied along both the ML and AP axes. Accordingly, in both the present and previous studies, the ML axis was the common direction of tactile stimulus movement. The moving tactile stimuli that induced phase-dependent body sway involved movement along the AP axis in the previous study, but not in the present study. Thus, those findings are explained by the view that the tactile stimuli moving along the ML axis phase dependently sway the body sway along the same axis, but this effect is cancelled by moving along the AP axis.

The change in body sway along the ML axis induced by the stimuli applied to the soles observed in the present study is consistent with previous findings indicating that a static change in the sensation of the soles induces COP displacement, particularly along the ML axis. For example, masking the tactile sensation of the soles increased the peak COP displacement to the left [8]. In addition, local anesthesia of the forefoot soles increased the COP area and velocity along the ML axis when vision was occluded [5]. Taken together, these results indicate that regardless of whether the change in the tactile sensation of the soles is static or dynamic, it specifically influences body sway along the ML axis. Body sway along the AP axis is mainly controlled by the ankle, while that along the ML axis is controlled by the hip [32]. Based on this, the phase-dependent change in body sway along the ML axis may reflect the fact that the tactile sensation of the soles contributes specifically to hip control during quiet stance.

Body sway with a dominant frequency below 1 Hz is generally attributed to control through the ankle strategy, while that above 1 Hz reflects control through the hip strategy [30]. Thus, to elucidate the effect of moving tactile stimuli on body sway along the ML axis, we measured the median frequency of COP displacement, which ranged from 0.2 to 0.4. This suggests that body sway was predominantly controlled through the ankle strategy. The frequency of COPy was significantly lower than that of COPx. Thus, the relatively greater contribution of the hip strategy to COPx may be the cause of the observed effect of moving tactile stimuli on body sway along the ML axis.

The COPx measured in phase 2 deviated significantly rightward compared with that in phases 8 and 9. Similarly, the value in phase 3 deviated significantly rightward compared with that in phase 9, as did that in phase 4. The loci of the tactile stimuli in phases 2, 3, and 4 were relatively leftward compared with those in phases 8 and 9. Accordingly, the body swayed in the direction contralateral to the stimulated side along the ML axis when tactile stimuli moving along the ML axis were applied. This postural response whereby the body sways opposite to the loci of tactile stimuli on the sole was observed in previous studies, where posture sway showed an opposite relationship with the area to which static tactile stimuli were applied [29,31]. In another previous study, participants maintained a quiet stance with the forefeet placed on a 6 × 8 pin matrix [33]. The movable plate supporting the pin matrix was driven by a servomotor, resulting in cyclic changes in the indentation of the plantar skin, thus producing a controlled perception of alternating increases and decreases in plantar pressure. When the pins moved at the forefoot, the COP was displaced in the opposite (posterior) direction relative to the maximum indentation. Accordingly, the present finding can be explained by the fact that humans perceive body sway toward the stimulated site and compensate for it by swaying the body towards the contralateral side.

### 4.3. Intermodal Reweighting

In a previous study, cutaneous anesthesia to the soles increased the displacement and velocity of the COP during quiet stance only when vision was occluded [5]. This indicates that humans rely on the cutaneous sensation of the soles for controlling body sway during quiet stance when vision is occluded. The loss of auditory cues increased the reliance of postural control on vision during quiet stance [34]. These findings are likely explained by the intermodal reweighting of vision and the other modalities of sensation [1,35,36,37,38]. Therefore, the tactile sensation of the soles likely contributes more to body sway control when vision is occluded.

Contrasting findings regarding the intermodal reweighting of vision and moving tactile sensation of the soles have been found. Previously, it was shown that the application of moving tactile stimuli mimicking the weight distribution change in the soles during gait did not change the total COP displacement [17]. In contrast, in the present study, the total COP displacement during moving tactile stimuli along the AP axis was larger than that measured under conditions of no stimuli and occluded vision. In the previous study, the tactile stimuli moved along both the AP and ML axes. In contrast, we observed a significant effect of moving tactile stimuli along the AP axis but an insignificant one under stimuli along the ML axis on total COP displacement when vision was occluded. Thus, the greater contribution of moving tactile stimuli to body sway caused by visual occlusion is due to the moving nature of the stimulus, particularly along the AP axis. This is explained by the fact that when vision is occluded, tactile stimuli moving particularly along the AP axis affect body sway control, but those moving along the ML axis cancel this effect.

Previous studies have demonstrated that visual occlusion makes a greater contribution to body sway along the AP axis than along the ML axis. For example, the standard deviation of the body sway of the waist and legs along the AP axis was greater when vision was occluded [39]. Similarly, the standard deviation of the COP displacement along the AP axis during quiet stance under visual occlusion conditions was greater than without visual occlusion [40]. Visual occlusion increased the peak of COP displacement, particularly along the AP axis [8]. Accordingly, the contribution of vision to the control of body sway is greater along the AP axis. Based on this, under occluded vision condition, the reduction in the contribution of vision to body sway control is greater for this axis; through the mechanism of intermodal reweighting, this reduction is compensated for with an increase in the contribution of tactile sensation to the control of body sway along the AP axis.

Visual occlusion increased the total COP displacement under stimuli along either the ML or AP axis, but this increase was not observed when the stimuli were not administered. This is likely because moving tactile sensation masked the cutaneous sensation of the soles, thus reducing the contribution of tactile sensation to body sway control. In this case, the contribution of vision to body sway control increased because of the intermodal reweighting of vision and tactile sensation. Thus, it is concluded that the increase in the COP displacement induced by visual occlusion, particularly during moving tactile stimuli, is due to the greater contribution of vision to body sway control when tactile sensation is masked by moving tactile stimuli due to the intermodal reweighting of vision and tactile sensation.

As shown above, because of intermodal reweighting, the contribution of tactile sensation to the control of body sway along the AP axis increases to compensate for the reduced contribution of vision to the control of COP displacement along this axis. On the one hand, the application of moving tactile stimuli along the ML axis induced a phase-dependent change in COPx under occluded vision conditions. This means that the contribution of moving tactile stimuli along the ML axis to the control of body sway along the same axis becomes apparent particularly when the contribution of vision to body sway control is absent. On the other hand, there were no effects of moving tactile stimuli along the AP axis, regardless of the vision condition. The mechanism of the intermodal reweighting of vision and tactile sensation particularly on the AP axis reported in previous studies seems to conflict with the phase-dependent effect of tactile stimuli, particularly along the ML axis, observed under visual occlusion condition in the present study. One possible explanation is that the phase-dependent body sway along the ML axis induced by phase-dependent change in the loci of tactile sensation along the same axis becomes apparent under occluded vision condition, and this mechanism is different from intermodal reweighting.

### 4.4. Mean COP

It was previously observed that the application of moving tactile stimuli mimicking the weight distribution change during gait did not change the mean COP [17], and this was consistent with the present finding. We calculated the mean COP based on the COP measurements during a session comprising 40 cycles, with the vibration devices being activated once per cycle. Thus, when averaging the COP over a session, the total time of tactile stimuli at each site was equal across the loci, although the timing of the stimulus at each locus varied. This must be why we observed no changes in the mean COP in the present study.

### 4.5. Limitations

The perception of tactile stimuli sometimes weakens over time because of adaptation [41]. Thus, the observed absence of effects of moving tactile stimuli along the AP axis may be due to the adaptation of tactile sensation to stimuli over time. Comparing the effects across different blocks of stimulation cycles within a session may be one way to examine this hypothesis. However, the number of cycles in each session was too small to be divided into several blocks. Thus, further studies are needed to examine this hypothesis.

The perception of the tactile stimuli produced by each vibrator may not have been the same across the devices. Thus, we cannot rule out the possibility that the variable perception of tactile stimuli among the vibrators may have influenced the findings.

In the present study, high-frequency vibration was applied for the selective activation of the cutaneous receptors rather than the muscle spindles [28,29]. However, the cutaneous receptors sensitive to high-frequency vibration are Pacinian corpuscles, which are not responsive to objects moving across the skin [25,26,27]. Therefore, the vibration frequency used in the present study may not have been optimal for activating skin receptors sensitive to moving tactile stimuli.

In the present study, the vibrator that was briefly actuated was switched from one unit to another to shift the stimulus location on the sole. However, a previous study has suggested that generating a sensation of moving touch through multipoint pattern displays is difficult because tactile receptors rapidly adapt [42]. To address this issue, Israr and Poupyrev proposed the Tactile Brush algorithm, which produces smooth moving tactile sensations by combining apparent tactile stimulation with phantom tactile illusions. In future studies, such methodologies may be applied to generate moving tactile sensation.

Although COP displacement is a widely accepted and sufficient measure for evaluating postural control during quiet stance, the present study did not include additional physiological or kinematic measurements such as electromyography, electroencephalography, or motion tracking. The absence of these multimodal recordings limits the extent to which the underlying neuromuscular and cognitive mechanisms of the observed body sway can be interpreted. Future studies incorporating such measures would allow for a more comprehensive assessment of the mechanical and neurophysiological processes associated with moving tactile stimuli to the soles.

### 4.6. Future Studies

Difficulty in gait initiation caused by gait freezing can occur in patients with Parkinson’s disease [43]. A previous study has shown that shifting weight to the initial stance side in advance causes better propulsion force of the stance leg in this population [44,45]. Anticipatory postural adjustment is weakened in patients with Parkinson’s disease, which may be a cause of the difficulty in gait initiation [46]. In healthy humans, rhythmically shifting weight between the feet causes better anticipatory postural adjustment for gait initiation [47]. Accordingly, the change in weight distribution in accordance with the phase of moving tactile stimuli along the ML axis may be a solution to treat the difficulty in gait initiation in patients with Parkinson’s disease. Therefore, clinical studies examining the effect of the plantar application of moving tactile stimuli in patients with Parkinson’s disease based on the results of the present study are warranted.

## 5. Conclusions

COP displacement along the ML axis was dependent on the phase of moving tactile stimuli along this axis under occluded vision condition. Thus, when humans perceive body sway along the ML axis, they compensate for it by displacing the body in the direction contralateral to the stimulated side. The greater effect of moving tactile stimuli on phase-dependent body sway, particularly under occluded vision condition, can be explained by the fact that the body sway along the ML axis influenced by the plantar loci receiving input of tactile sensation moving along the same axis becomes apparent under occluded vision condition. The total COP displacement during tactile stimuli along the AP axis was larger than that without stimuli, particularly under occluded vision condition. This can be explained by the fact that, because of intermodal reweighting, the contribution of tactile sensation to the control of body sway along the AP axis increases to compensate for the lack of visual input regulating COP displacement along this axis.

## Data Availability

The original contributions presented in this study are included in the article. Further inquiries can be directed to the corresponding author(s).

## References

[1] Chiba R., Takakusaki K., Ota J., Yozu A., Haga N. (2016). Human upright posture control models based on multisensory inputs; in fast and slow dynamics. Neurosci. Res..

[2] Viseux F., Lemaire A., Barbier F., Charpentier P., Leteneur S., Villeneuve P. (2019). How can the stimulation of plantar cutaneous receptors improve postural control? Review and clinical commentary. Neurophysiol. Clin..

[3] Perry S.D. (2006). Evaluation of age-related plantar-surface insensitivity and onset age of advanced insensitivity in older adults using vibratory and touch sensation tests. Neurosci. Lett..

[4] Maitre J., Paillard T. (2016). Influence of the Plantar Cutaneous Information in Postural Regulation Depending on the Age and the Physical Activity Status. Front. Hum. Neurosci..

[5] Meyer P.F., Oddsson L.I., De Luca C.J. (2004). The role of plantar cutaneous sensation in unperturbed stance. Exp. Brain Res..

[6] Wang T.Y., Lin S.I. (2008). Sensitivity of plantar cutaneous sensation and postural stability. Clin. Biomech..

[7] Turcot K., Allet L., Golay A., Hoffmeyer P., Armand S. (2009). Investigation of standing balance in diabetic patients with and without peripheral neuropathy using accelerometers. Clin. Biomech..

[8] Sawaguchi Y., Kawasaki T., Oda H., Kunimura H., Hiraoka K. (2022). Contribution of vision and tactile sensation on body sway during quiet stance. J. Phys. Ther. Sci..

[9] Lauzier L., Kadri M.A., Bouchard E., Bouchard K., Gaboury S., Gagnon J.M., Girard M.P., Larouche A., Robert R., Lapointe P. (2021). Vibration of the whole foot soles surface using an inexpensive portable device to investigate age-related alterations of postural control. Front. Hum. Neurosci..

[10] Pei Y.C., Bensmaia S.J. (2014). The neural basis of tactile motion perception. J. Neurophysiol..

[11] Amemiya T., Beck B., Walsh V., Gomi H., Haggard P. (2017). Visual area V5/hMT+ contributes to perception of tactile motion direction: A TMS study. Sci. Rep..

[12] Bremmer F., Schlack A., Shah N.J., Zafiris O., Kubischik M., Hoffmann K.P., Fink G.R. (2001). Polymodal motion processing in posterior parietal and premotor cortex: A human fMRI study strongly implies equivalencies between humans and monkeys. Neuron.

[13] Hagen M.C., Franzén O., McGlone F., Essick G., Dancer C., Pardo J.V. (2002). Tactile motion activates the human middle temporal/V5 (MT/V5) complex. Eur. J. Neurosci..

[14] Summers I.R., Francis S.T., Bowtell R.W., McGlone F.P., Clemence M. (2009). A functional-magnetic-resonance-imaging investigation of cortical activation from moving vibrotactile stimuli on the fingertip. J. Acoust. Soc. Am..

[15] Wacker E., Spitzer B., Lützkendorf R., Bernarding J., Blankenburg F. (2011). Tactile motion and pattern processing assessed with high-field FMRI. PLoS ONE.

[16] Van Kemenade B.M., Seymour K., Wacker E., Spitzer B., Blankenburg F., Sterzer P. (2014). Tactile and visual motion direction processing in hMT+/V5. Neuroimage.

[17] Sawaguchi Y., Kawasaki T., Hiraoka K. (2023). Effect of Moving Tactile Stimuli to Mimic Altered Weight Distribution During Gait on Quiet Stance Body Sway. Percept. Mot. Skills.

[18] Hamill P.V., Drizd T.A., Johnson C.L., Reed R.B., Roche A.F. (1977). NCHS Growth Curves for Children Birth-18 Years.

[19] Thomas J.R., French K.E. (1985). Gender differences across age in motor performance: A meta-analysis. Psychol. Bull..

[20] Gribble P.A., Robinson R.H., Hertel J., Denegar C.R. (2009). The effects of gender and fatigue on dynamic postural control. J. Sport Rehabil..

[21] Kahraman B.O., Kahraman T., Kalemci O., Sengul Y.S. (2018). Gender differences in postural control in people with nonspecific chronic low back pain. Gait Posture.

[22] Elias L.J., Bryden M.P., Bulman-Fleming M.B. (1998). Footedness is a better predictor than is handedness of emotional lateralization. Neuropsychologia.

[23] Zverev Y.P. (2006). Spatial parameters of walking gait and footedness. Ann. Hum. Biol..

[24] Kunimura H., Oda H., Kawasaki T., Tsujinaka R., Hamada N., Fukuda S., Matsuoka M., Hiraoka K. (2023). Effect of Laterally Moving Tactile Stimuli to Sole on Anticipatory Postural Adjustment of Gait Initiation in Healthy Males. Brain Sci..

[25] Talbot W.H., Darian-Smith I., Kornhuber H.H., Mountcastle V.B. (1968). The sense of flutter-vibration: Comparison of the human capacity with response patterns of mechanoreceptive afferents from the monkey hand. J. Neurophysiol..

[26] Johansson R.S., Landström U., Lundström R. (1982). Responses of mechanoreceptive afferent units in the glabrous skin of the human hand to sinusoidal skin displacements. Brain Res..

[27] Bolanowski S.J., Gescheider G.A., Verrillo R.T., Checkosky C.M. (1988). Four channels mediate the mechanical aspects of touch. J. Acoust. Soc. Am..

[28] Roll J.P., Vedel J.P. (1982). Kinaesthetic role of muscle afferents in man, studied by tendon vibration and microneurography. Exp. Brain Res..

[29] Kavounoudias A., Roll R., Roll J.P. (1998). The plantar sole is a ‘dynamometric map’ for human balance control. Neuroreport.

[30] Creath R., Kiemel T., Horak F., Peterka R., Jeka J. (2005). A unified view of quiet and perturbed stance: Simultaneous co-existing excitable modes. Neurosci. Lett..

[31] Kavounoudias A., Roll R., Roll J.P. (2001). Foot sole and ankle muscle inputs contribute jointly to human erect posture regulation. J. Physiol..

[32] Winter D.A., Prince F., Sterior P. (1993). Medial-lateral and anterior-posterior motor responses associated with center of pressure changes in quiet standing. Neurosci. Res. Commun..

[33] Maurer C., Mergner T., Bolha B., Hlavacka F. (2001). Human balance control during cutaneous stimulation of the plantar soles. Neurosci. Lett..

[34] Maurer C., Mergner T., Peterka R.J. (2006). Multisensory control of human upright stance. Exp. Brain Res..

[35] Peterka R.J. (2002). Sensorimotor integration in human postural control. J. Neurophysiol..

[36] Peterka R.J. (2018). Sensory integration for human balance control. Handbook of Clinical Neurology.

[37] Maheu M., Sharp A., Landry S.P., Champoux F. (2017). Sensory reweighting after loss of auditory cues in healthy adults. Gait Posture.

[38] Assländer L., Peterka R.J. (2016). Sensory reweighting dynamics following removal and addition of visual and proprioceptive cues. J. Neurophysiol..

[39] Dickstein R., Abulaffio N. (2000). Postural sway of the affected and nonaffected pelvis and leg in stance of hemiparetic patients. Arch. Phys. Med. Rehabil..

[40] Carpenter M.G., Frank J.S., Silcher C.P., Patla A.E. (2001). The influence of postural threat on the control of upright stance. Exp. Brain Res..

[41] Wark B., Lundstrom B.N., Fairhall A. (2007). Sensory adaptation. Curr. Opin. Neurobiol..

[42] Israr A., Poupyrev I. Tactile brush: Drawing on skin with a tactile grid display. Proceedings of the SIGCHI Conference on Human Factors in Computing Systems.

[43] Palmisano C., Brandt G., Vissani M., Pozzi N.G., Canessa A., Brumberg J., Marotta G., Volkmann J., Mazzoni A., Pezzoli G. (2020). Gait Initiation in Parkinson’s Disease: Impact of Dopamine Depletion and Initial Stance Condition. Front. Bioeng. Biotechnol..

[44] Azuma T., Ito T., Yamashita N. (2007). Effects of changing the initial horizontal location of the center of mass on the anticipatory postural adjustments and task performance associated with step initiation. Gait Posture.

[45] Caderby T., Yiou E., Peyrot N., De Vivies X., Bonazzi B., Dalleau G. (2017). Effects of changing body weight distribution on mediolateral stability control during gait initiation. Front. Hum. Neurosci..

[46] Burleigh-Jacobs A., Horak F.B., Nutt J.G., Obeso J.A. (1997). Step initiation in Parkinson’s disease: Influence of levodopa and external sensory triggers. Mov. Disord..

[47] Hiraoka K., Kunimura H., Oda H., Kawasaki T., Sawaguchi Y. (2020). Rhythmic movement and rhythmic auditory cues enhance anticipatory postural adjustment of gait initiation. Somatosens. Mot. Res..

